# Impact of intrafraction fiducial tracking on planning target margins during prostate stereotactic body radiotherapy using Brainlab ExacTrac Dynamic with robotic couch correction

**DOI:** 10.1002/acm2.70363

**Published:** 2025-11-18

**Authors:** Matthew Worth, Daniel C. Brancato, Julie Lo, Thomas P. Kole

**Affiliations:** ^1^ Department of Radiation Oncology The Valley Hospital One Valley Health Plaza Paramus New Jersey USA

**Keywords:** brainlab ExacTrac dynamic, fiducial‐based image guidance, intrafraction motion management, planning target volume (PTV) margin reduction, prostate cancer, six‐degree‐of‐freedom (6DoF) couch correction, stereotactic body radiotherapy (SBRT)

## Abstract

**Background:**

Planning target volume (PTV) margin reduction has been demonstrated to result in decreased acute genitourinary and gastrointestinal toxicity in patients treated with definitive stereotactic body radiotherapy (SBRT) for localized prostate cancer.

**Purpose:**

The purpose of this study was to assess the impact of stereoscopic x‐ray‐based intrafraction prostate fiducial tracking with six degrees of freedom (6DoF) robotic couch correction on PTV margin reduction during gantry‐based prostate SBRT.

**Materials and Methods:**

Intrafraction prostate position data was analyzed for patients with localized prostate cancer treated using gantry‐based SBRT with stereoscopic x‐ray intrafraction fiducial monitoring using Brainlab ExacTrac Dynamic (ETD) and 6DoF couch correction at our institution. Fiducial displacements and rotations were calculated between each intrafraction measurement, and mean intrafraction deviations were determined for each fraction, patient, and the overall cohort. Minimum PTV margins required in each dimension were calculated using the van Herk method.

**Results:**

There were 2525 intrafraction stereoscopic fiducial verifications within the cohort. Intrafraction fiducial displacements were within 1.5 mm between 98.7%, 96.1%, and 95.1% consecutive fiducial verifications in the left/right (LR), superior/inferior (SI), and anterior/posterior (AP) dimensions, respectively. Rotational deviations were within 2° between 83.3%, 98.3%, and 99.4% consecutive fiducial verifications in the pitch, roll, and yaw directions, respectively. Twenty‐three percent of stereoscopic verifications triggered a robotic couch correction based upon specified tolerances. Overall, 56% of treatment fractions required a 6DoF couch correction. Fiducial position verifications requiring couch correction were most often triggered by pitch, which occurred in 72% of instances. This corresponded to required minimum PTV margins of 1.23, 1.73, and 1.44 mm in the LR, SI, and AP dimensions, respectively.

**Conclusions:**

Intrafraction fiducial tracking using ETD stereoscopic x‐ray verification with 6DoF robotic couch correction allows for reduced PTV margins when treating localized prostate cancer with gantry‐based SBRT.

## 1 INTRODUCTION

Stereotactic body radiotherapy (SBRT) is a standard curative treatment option for organ‐confined prostate cancer.^1^ In the United States, prostate SBRT typically involves the delivery of five high‐dose radiation treatments to the prostate over 1–2 weeks. Delivery of high dose per fraction radiation utilized in prostate SBRT is technologically challenging due to intrafraction motion of the prostate, which is influenced by external forces exerted by the adjacent bladder and rectum.^2^ Due to this motion, planning target volume (PTV) margins around the prostate are required to ensure adequate dose coverage of the clinical target volume (CTV). Unfortunately, these margins result in increasing volumes of adjacent normal tissue that receive high doses of radiation^3^ and subsequently an increase in the risk of treatment‐related toxicity.^4^ Accordingly, improved localization of the prostate during SBRT is critical to minimize necessary PTV margins and therefore decrease the risk of injury to adjacent dose‐limiting critical organs such as the bladder and rectum by reducing the volumes of these tissues exposed to radiation during treatment.^5^ It is important that PTV margin reduction is specific to the technology used for prostate localization and motion management given that PTV margin reduction beyond the capabilities of motion capture may result in treatment failure.^6^

Treatment margins applied in radiation therapy must account for both systematic and random errors associated with treatment setup and delivery. Methods for determining adequate radiation treatment margins have been previously described by van Herk, using positional data collected from large numbers of patients treated with various radiation treatment imaging methods.^7^ Levin‐Epstein et al. previously applied this method to patients with localized prostate cancer treated with SBRT using kV or MV orthogonal x‐ray‐based image‐guided radiation therapy (IGRT) of implanted radio‐opaque prostate fiducials.^8^ Despite limited real‐time imaging capabilities without rotational couch corrections, their analysis of intrafraction motion indicated necessary minimum prostate margins of 1.9, 2.7, and 3.1 mm in the lateral, superior/inferior, and anterior/posterior directions, respectively.

As imaging technology continues to improve, more aggressive prostate PTV margin reduction has been made possible. Real‐time MRI linear accelerators (MRL) have emerged as a promising new means of prostate localization and motion management during prostate radiation therapy. A recent randomized trial of 156 men treated with MRL or cone beam CT (CBCT)‐based SBRT for prostate cancer utilized an unprecedented 2 mm margin around the prostate for patients treated with MRL SBRT compared to a 4 mm margin for CBCT‐based SBRT. This resulted in decreased acute urinary and bowel‐related toxicity in patients receiving MRL prostate SBRT.^9^ Unfortunately, MRL is expensive technology that requires special considerations, which may not be feasible or practical for most clinics with established gantry‐mounted linear accelerators. Furthermore, it remains to be determined whether this aggressive margin reduction will have any long‐term impact on treatment efficacy or chronic toxicity.

Upgrading existing linear accelerators with advanced intrafraction imaging capabilities may be a more economical approach to improve radiation treatment accuracy and confidently reduce PTV margins during prostate SBRT. Brainlab ExacTrac Dynamic (ETD) is an off‐axis, paired x‐ray‐based IGRT system that also incorporates real‐time optical and thermal surface imaging to monitor a patient's position during radiation treatment. This system has demonstrated similar positional accuracy to static CT imaging; however, with the additional benefit of real‐time six degree of freedom (6DoF) couch corrections during treatment delivery.^10^ The aim of this study was to examine the impact of the ETD IGRT system with 6DoF robotic positioning on prostate motion detection and perform a systematic spatial and temporal analysis to determine appropriate prostate treatment margins in men with organ‐confined prostate cancer undergoing treatment with prostate SBRT using this technology.

## 2 METHODS

This study was conducted on a retrospective institutional protocol granted IRB exemption. Data from patients treated for organ‐confined prostate cancer using SBRT at our institution following the installation of ETD were included in this analysis. All treatments were delivered on Varian TrueBeam or Edge gantry‐based linear accelerators with PerfectPitch 6DoF robotic couches (Varian Medical Systems, CA) and ETD advanced imaging.

### 2.1 Treatment simulation

**FIGURE 1:**
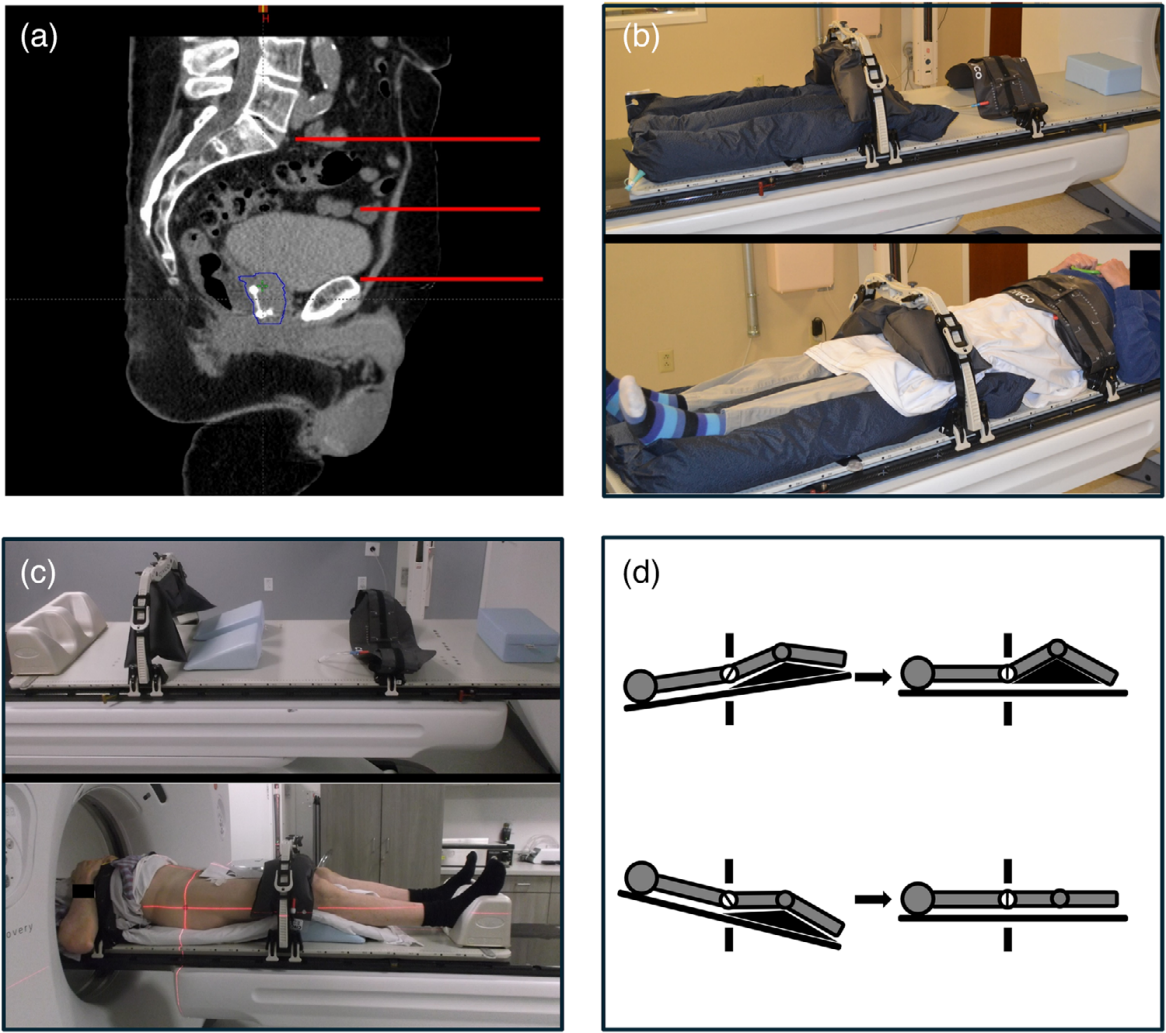
Prostate SBRT simulation and treatment setup. (a) Ideal patient bladder fill with the superior most extent of the bladder indicated by the middle red line located approximately half the distance between the top of the pubic symphysis and the sacral promontory. (b) Fixed prostate SBRT immobilization setup. (c) Adapted adjustable prostate SBRT setup. (d) Prostate pitch adjustment using different knee positioning.

Patients were implanted with two FlexiMarc G/T 1.2 mm diameter, 2 node, 20 mm spacing, gold‐on‐titanium fiducial markers preloaded in sterile 17Ga × 20 cm needles (Innovative Oncology Solutions, TN) along with a temporary rectal hydrogel spacer (SpaceOAR, Boston Scientific, MA) approximately 7 days prior to CT simulation and high‐resolution (1 mm axial slices) T2 MRI of the prostate. On the day of simulation, patients were instructed to perform a Fleet enema 2 h prior to their appointment to empty their rectum and then maintain a semi‐full bladder, which was achieved by emptying of the bladder approximately 45 min prior to imaging followed by ingestion of 8–16 oz of water. Bladder fullness was evaluated during simulation using a modification of the method described by Kim et al., where the most superior extent of the bladder was targeted to extend superior to the pubic symphysis by approximately 40%–60% of the distance between the top of the pubic symphysis and sacral promontory (Figure 1a).^11^

Patients treated with prostate SBRT at our institution were traditionally immobilized using rigid fixation consisting of a large Vac‐Lok cradle (Innovative Oncology Solutions, TN) from the lower pelvis through the upper torso, with multiple compressive immobilizers extending from the upper abdomen to the feet (Figure 1b). This was based upon our previous experience using CBCT‐guided prostate SBRT with limited real‐time intrafraction motion management. Given the additional optical and thermal surface imaging capabilities of ETD to monitor intrafraction patient motion, we transitioned to a more comfortable and flexible immobilization, which consisted of a head‐first supine setup on a thin mat with 2 × 20° wedges (Civco Medical Solutions, IA) placed under the knees (Figure 1c). This also allowed for flexibility in knee positioning during treatment sometimes needed to account for rotational deviations beyond the 3° limitations of the 6Dof couch. For instance, at the start of each treatment fraction, if the observed fiducial pitch deviation exceeded −3° (head down), then a larger single 45° knee wedge (Civco Medical Solutions, IA) was placed under the knees to neutralize the negative pitch. Conversely, if the pitch exceeded +3° (feet down), the initial wedges were removed to neutralize the positive pitch (Figure 1d).

### 2.2 Treatment planning

High‐resolution T2 MRI images of the prostate were registered to the planning simulation CT images in Eclipse v15.6 (Varian Medical Systems, CA) treatment planning system using a combination of automatic and manual fusion based on fiducial alignment. The CTV was defined as the prostate and the proximal 1–2 cm of the seminal vesicles. The PTV consisted of the CTV with a uniform 3 mm radial expansion. Plans were generated using Varian's Photon Optimizer and Anisotropic Analytical Algorithm to deliver a dose of 36.25 Gy in five fractions to the PTV with or without an integrated boost of 40 Gy to the CTV using a RapidArc (Varian Medical Systems, CA) intensity modulated radiation therapy (IMRT) approach with 6 or 10 MV photons in flattening filter free (FFF) mode. Each treatment plan consisted of 2–4 arcs. Approved treatment plans were exported to the ETD application and prepared using the preparation workspace. The ETD implanted marker workflow, based on automatic detection and registration of fiducials, was selected as the target localization method for patient positioning. An institutional prostate SBRT‐specific template was attached to each treatment plan. The “short markers” option was selected to annotate the individual fiducials. The center of each fiducial was manually defined by aligning all planes on the reconstructed CT to the center of the fiducial and placing the location at the intersection.

### 2.3 ETD quality assurance

Daily and monthly quality assurance (QA) testing of the ETD stereoscopic x‐ray, optical, and thermal imaging systems was completed according to manufacturers’ recommendations to ensure accuracy and reproducibility. Daily QA consisted of offsetting a Brainlab QA phantom by a specified distance from the linear accelerator isocenter. ETD images were taken of the phantom and analyzed to determine the deviation between the surface thermal camera and the x‐ray positioning unit and the deviation from the last calibration radiation isocenter. Finally, a shift of the phantom was performed to verify initial specified offset and repositioning accuracy. Failure of the system to reposition the phantom to linac isocenter or achieve all the following requires system recalibration: translational deviation consistency vector length less than 1 mm, rotational deviation consistency of each component less than 1°, reconstruction error less than or equal to 0.09 mm, and an average projection error of the x‐ray markers less than or equal to 0.7 pixels.

Each month, the ETD thermal camera was recalibrated using the BrainLab Thermal to 3D Calibration Phantom. A series of x‐ray verification image acquisitions were also performed to reestablish a baseline for detector functionality. After the thermal camera calibration and x‐ray verifications, a Winston Lutz test was performed with IsoCal/Iso Verification (Varian, CA) for linear accelerator isocenter verification (<0.5 mm vector). Following verification of the machine isocenter and corresponding laser adjustment, the BrainLab QA phantom was used to complete the BrainLab surface and radiation isocenter calibration as described above to ensure accuracy within 0.5 mm vector.

### 2.4 Treatment delivery

For each treatment, patients were initially aligned externally using surface contours shown on ETD and tattoos. This was followed by a CBCT to verify appropriate bowel and bladder fill and to perform an initial prostate fiducial registration. Couch corrections were applied accordingly. Additional rotational adjustments outside of the capabilities of the 6DoF couch were performed by patient adjustment as described above. Before the start of each arc of therapy, stereoscopic x‐ray verifications were performed using the ETD‐implanted marker workflow with automated prostate fiducial detection and any identified couch corrections were applied. Continuous surface monitoring was performed by ETD with allowable surface translational tolerances of 5 mm in the left/right (LR), superior/inferior (SI), and anterior/posterior (AP) dimensions, and rotational tolerances of 5° for pitch, roll, and yaw. Automated stereoscopic x‐ray verifications were performed at each of the cardinal gantry angles (0°, 90°, 180°, and 270°) with permissible x‐ray tolerances of 1.5 mm for translations and 2° for rotations. Measured prostate fiducial deviations exceeding any of these tolerances triggered an auto‐beam stop for application of couch corrections.

### 2.5 Prostate motion analysis

All data analysis was performed using custom MATLAB (MathWorks Inc., MA) scripts. Prostate motion was assessed using calculated prostate fiducial displacements in the LR, SI, and AP dimensions. Intrafraction fiducial displacements were calculated between each prostate fiducial measurement during every treatment for all patients. Mean displacements $\left(\bar{d}\right)$ for each patient were calculated by averaging the intrafraction displacements over the full course of treatment in each dimension. The dimensional standard deviations ($\bar{s}$) for the overall cohort were calculated from the intrafraction mean displacements for each patient.

The method previously described by van Herk and applied to prostate motion by Levin‐Epstein et al. was used to calculate the PTV margins necessary in each dimension to ensure that the CTV received at least 95% of the prescription dose of radiation for 90% of patients.^7^, ^8^ This method is exclusively based on translational deviations and does not account for rotational errors and target shape deviations.

margin=2.5Σ+0.7σ

Here *Σ* indicates the standard deviation of the systematic error and *σ* is the standard deviation of the random error associated with prostate fiducial position. For intrafraction prostate motion, *Σ* is approximated by $\bar{s}$ and *σ* is calculated from the root mean square of the standard deviations of intrafraction prostate fiducial shifts for each patient.

## 3 RESULTS

**FIGURE 2:**
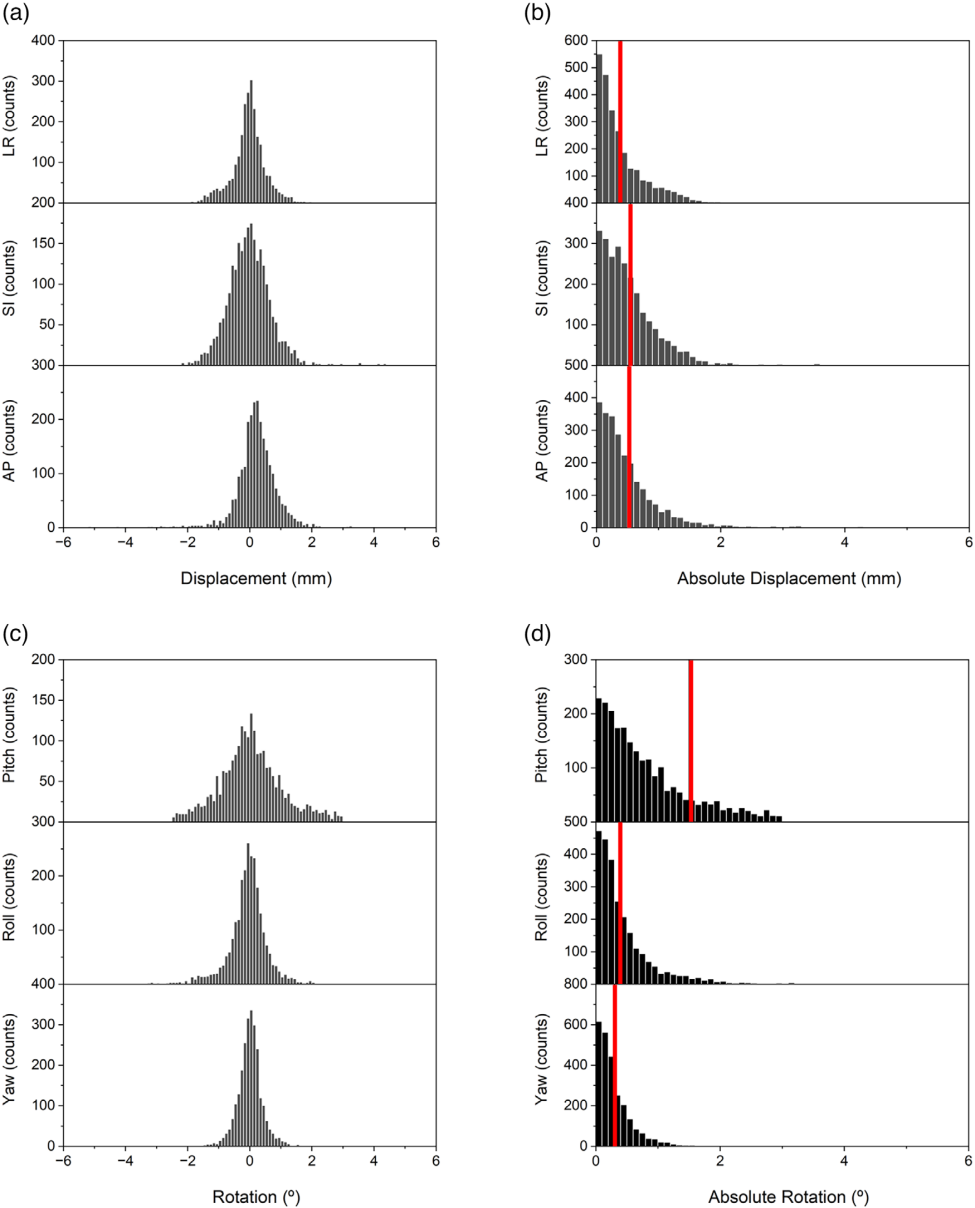
Distribution of intrafraction prostate fiducial deviations. Histogram plots of translational (a and b) and rotational (c and d) movements between intrafraction stereoscopic x‐ray fiducial verifications. Directional distribution plots are shown in (a) and (c), and absolute distributions are shown in (b) and (d). Mean absolute translational and rotational displacements are indicated by the red line (b and d).

ETD prostate fiducial tracking data was available for 76 patients over five fractions of prostate SBRT yielding 2525 intrafraction fiducial verifications for analysis. Individual translational displacements between stereoscopic x‐rays were distributed around means of 0.03, 0, and 0.2 mm in the LR, SI, and AP dimensions with standard deviations of 0.54, 0.8, and 0.79 mm, respectively (Figure 2a). This translated into absolute mean LR, SI, and AP fiducial displacements between stereoscopic x‐ray images of 0.38, 0.55, and 0.53 mm (Figure 2b). Similarly, individual rotations were distributed around means of 0.07°, 0.07°, and 0.02° in the pitch, roll, and yaw directions with standard deviations of 1.78°, 0.65°, and 0.46°, respectively (Figure 2c). Ultimately, this resulted in absolute mean rotational pitch, roll, and yaw deviations of 1.2°, 0.4°, and 0.3° (Figure 2d).

**FIGURE 3:**
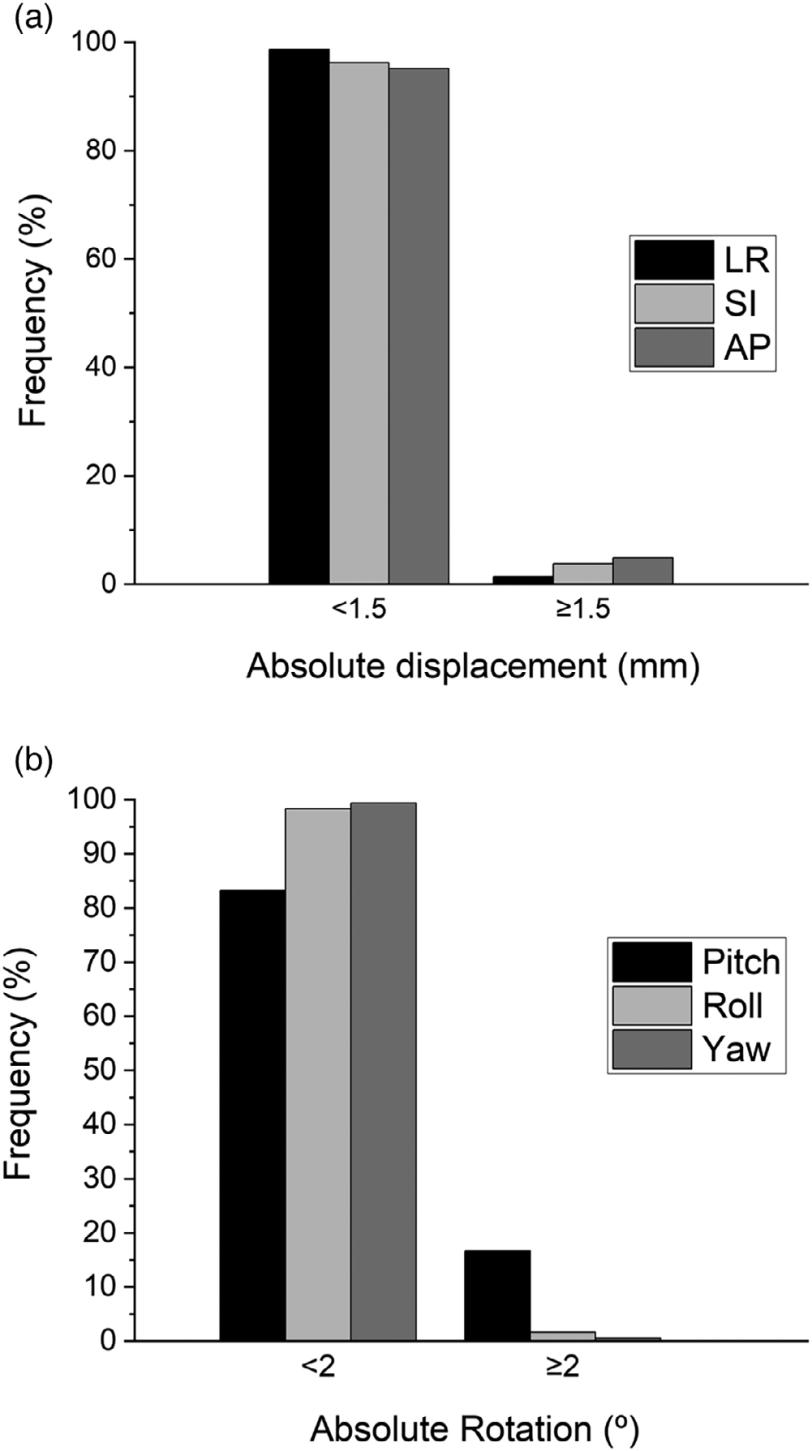
Prostate motion tolerance. Overall frequency of stereoscopic fiducial x‐ray verifications within or exceeding 1.5 mm translational (a) and 2° rotational (b) tolerances.

**FIGURE 4:**
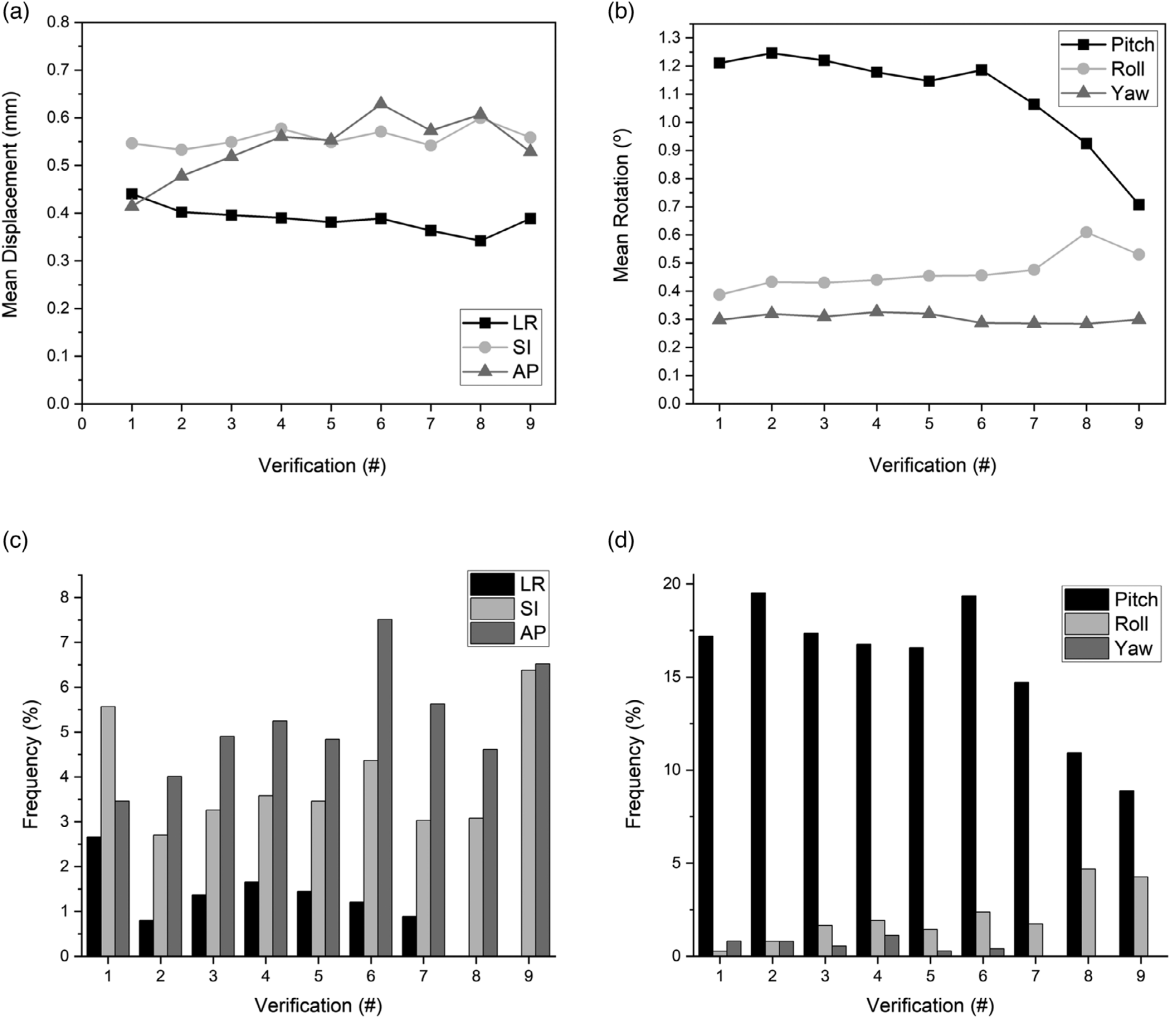
Prostate motion over time. Mean treatment fraction translational (a) and rotational (b) deviations as a function of stereoscopic fiducial x‐ray verification. Relative frequency of translational (c) and rotational (d) deviations exceeding tolerance as a function of stereoscopic fiducial x‐ray verification during each fraction.

Absolute translational fiducial displacements were within the specified 1.5 mm tolerance in 98.7%, 96.1%, and 95.1% of sequential stereoscopic x‐ray fiducial verifications in the LR, SI, and AP dimensions (Figure 3a). Larger excursions were infrequent with maximum translations of 2.65, 7.1, and 8.27 mm, respectively. Identified rotational deviations were within the 2° tolerance in 83.3%, 98.3%, and 99.4% of sequential fiducial verifications in the pitch, roll, and yaw dimensions (Figure 3b) with maximum rotations of 9.53°, 3.93°, and 4.36°, respectively. Examination of mean fiducial translations as a function of treatment verification number during each treatment fraction demonstrated mostly stable mean fiducial displacement over the duration of treatment fractions (Figure 4a). Mean displacements in the AP dimension appeared to increase slightly from verification 1 to verification 9 (0.41–0.53 mm), however, this was not statistically significant when analyzed by ANOVA with multiple comparisons (*p* > 0.05). Mean rotational deviations remained largely stable throughout treatment verifications. However, mean pitch rotations were greater than roll and yaw rotations and tended to decrease toward the end of each treatment fraction, although this trend was not statistically significant (Figure 4b). Additionally, the frequency of translational and rotational deviations exceeding specified tolerances was statistically similar (*p* > 0.05) among treatment verifications (Figure 4c,d).

**FIGURE 5:**
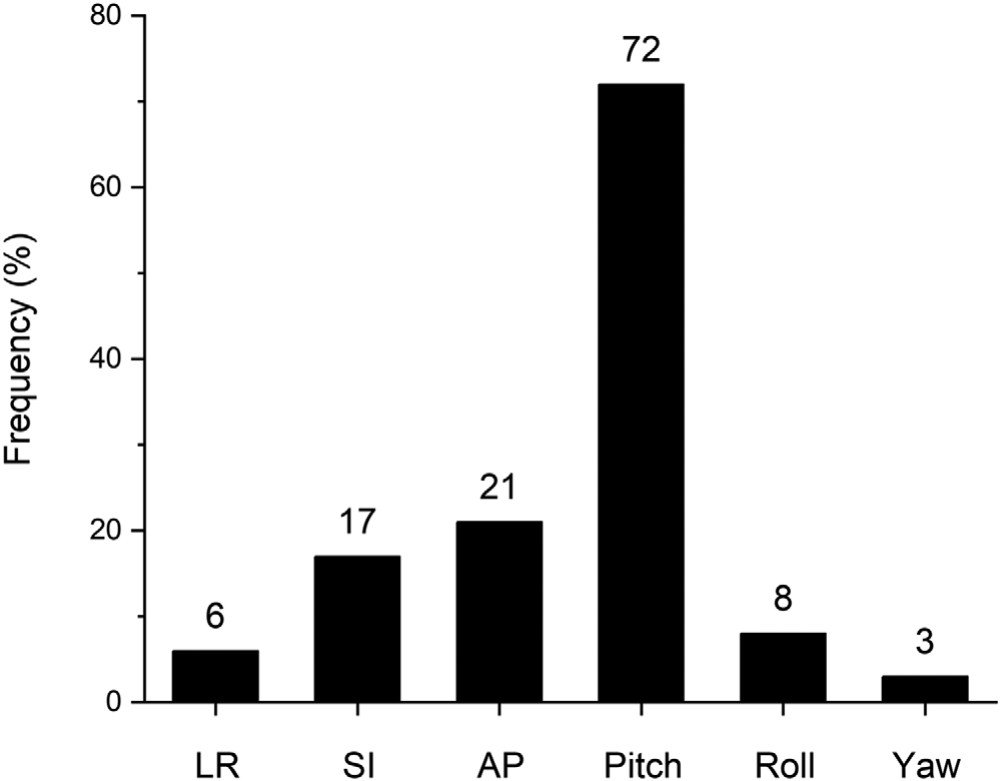
Source of triggered corrections. Frequency of translational and rotational deviations exceeding tolerance during stereoscopic fiducial x‐ray verifications that triggered an auto beam stop.

Overall, x‐ray triggered auto‐beam stops occurred in 23% of stereoscopic fiducial x‐ray verifications due to exceeding at least one translational or rotational tolerance. This resulted in 56% of treatment fractions requiring intrafraction couch adjustment. Rotational pitch corrections were most often encountered with 72% of triggered beam stops exceeding the 2° rotational threshold (Figure 5). Tolerance thresholds in the AP and SI dimensions were the other most common sources of x‐ray triggered auto‐beam stops occurring in 21% and 17% of triggered corrections, respectively.

Accordingly, systematic and random errors associated with intrafraction motion using the van Herk method were greatest in the SI dimension (Table 1). This resulted in calculated minimum required PTV margins to account for intrafraction prostate motion of 1.23, 1.73, and 1.44 mm in the LR, SI, and AP dimensions, respectively.

**TABLE 1:** PTV margin analysis.

	Distance (mm)
Systematic error (*Σ*)	
LR	0.38
SI	0.51
AP	0.37
Random error (*σ*)	
LR	0.41
SI	0.66
AP	0.72
PTV margin	
LR	1.23
SI	1.73
AP	1.44

*Note*: Systematic and random errors were determined from intrafraction stereoscopic fiducial x‐ray verifications for calculation of minimum prostate CTV to PTV margins using the van Herk method.

Abbreviations: AP, anterior/posterior; LR, left/right; PTV, planning target volume; SI, superior/inferior.

## 4 DISCUSSION

Intrafraction prostate motion is a known complicating factor in the treatment of prostate cancer using radiation therapy.^3^, ^8^, ^12^, ^13^, ^14^, ^15^, ^16^, ^17^ As prostate radiation treatment courses have become shorter by delivering higher radiation doses per fraction, excess PTV margins required to compensate for prostate motion have been recognized as a critical source of treatment‐related toxicity.^18^ This was recently demonstrated in the MIRAGE trial, where real‐time MRI‐based IGRT allowed for a decrease in prostate SBRT PTV margin expansion from 4 to 2 mm, resulting in significant reduction in cumulative grade 2 or greater genitourinary (27% vs. 51%) and gastrointestinal toxicity (1.4% vs. 9%).^9^, ^19^ Unfortunately, adoption of MRI‐guided linear accelerator technology requires a significant capital investment, which may not be practical for many radiation oncology facilities. Additionally, concerns with patient throughput limit general use feasibility of these accelerators.^20^

The results of our study suggest that aggressive PTV margin reduction in the delivery of prostate SBRT may be achieved using contemporary gantry‐based linear accelerators with enhanced intrafraction IGRT capabilities and 6DoF intrafraction couch correction. Using the ETD system, we found mean absolute fiducial translational displacements between consecutively acquired intrafraction stereoscopic x‐ray images that were less than 0.55 mm in all dimensions. Fiducial shifts in the SI and AP dimensions were typically larger than those in the LR dimension, consistent with previous reports of intrafraction prostate motion.^3^, ^13^, ^14^, ^16^, ^21^ Large intrafraction translational deviations were infrequent with deviations exceeding 1.5 mm occurring in only 5% of stereoscopic x‐ray fiducial verifications.

Using the van Herk method,^7^ the largest necessary margin we identified was 1.73 mm in the SI dimension, while margins of only 1.44 and 1.23 mm were required in the AP and LR dimensions, respectively. To our knowledge, this is the smallest reported required CTV‐to‐PTV margin for gantry‐mounted linear accelerator prostate SBRT using x‐ray‐based IGRT without the use of intracavitary fixation devices. Levin‐Epstein et al. previously conducted a study of intrafraction prostate motion using data collected from 205 patients treated on prospective clinical trials of gantry‐mounted prostate SBRT at a single institution.^8^ All patients had radio‐opaque prostate fiducials implanted prior to radiation treatment. Patients were imaged at the start of radiation treatment and then at three subsequent time points during each treatment using either MV or kV orthogonal x‐ray paired images. Intrafraction translational corrections were applied for measured shifts of >1 mm in any dimension. Using their approach, prostate PTV margins of 1.9, 2.7, and 3.1 mm in the LR, SI, and AP dimensions were required for adequate prescription dose coverage of the PTV. The nearly 1 and 1.7 mm improvements noted in our calculated SI and AP margins may be attributed to more frequent intrafraction imaging in the present study (every 90° of arc therapy instead of 180°), together with the measurement and application of both translational and rotational intrafraction couch corrections.

Our institutional policy is to correct all intrafraction translational deviations greater than 1.5 mm and rotational deviations greater than 2°. Additionally, deviations of any magnitude are corrected before the start of each treatment arc. Using this methodology, we found that auto beam stops occurred in 23% of intrafraction fiducial verifications, which is similar to the rate of 20.6% reported by Mangesius et al. using ETD‐based prostate fiducial intrafraction monitoring in the delivery of conventionally fractionated and moderately hypofractionated prostate radiotherapy.^22^ They noted that in the absence of intrafraction translational correction, prostate fiducial deviations exceeding 2 mm would have occurred in 39.4% of verifications. However, in their study translational corrections were applied for measured shifts greater than 2 mm, and although rotational deviations were measured during each fiducial verification, rotational couch corrections were not applied. We found that rotational deviations exceeding tolerance were more common than exceeded translational deviations, and most often occurred in the pitch direction. Similar trends for prostate rotational behavior were noted in the Mangesius study with identified pitch deviation angles of more than two‐fold yaw or roll.^22^

The duration of treatment delivery has been identified as an important factor in intrafraction prostate motion where the magnitude of prostate deviations from the initial point of origin has been shown to increase proportionally to the length of treatment duration along with greater frequency of large prostate excursions.^13^, ^14^, ^16^ This was not observed in our cohort as mean translational and rotational displacements and rates of displacements exceeding our specified tolerances remained consistent throughout the course of each fraction (Figure 4). This may be attributed to the shorter “beam on” time of FFF‐VMAT beams (4–6 min per fraction) and the robotic 6DoF couch corrections that occurred at the start of each arc of therapy, which effectively “re‐zeroed” the couch to the starting position. Additionally, our patient immobilization setup allowed for initial correction of excess prostate pitch deviations that exceeded the limitations of the 6DoF couches, essentially maximizing the capabilities of the robotic repositioning system. Lastly, it is possible that the viscoelastic properties of the hydrogel spacers implanted between the prostate and rectum for each patient acted as a dampener, effectively stabilizing the prostate. However, a previous analysis of real‐time prostate motion using electromagnetic transponders in patients with and without hydrogel spacers demonstrated similar patterns of intrafraction motion.^23^

The minimum CTV to PTV margins that we have derived are limited to those accounting for intrafraction prostate motion and are based on translational deviations only. However, errors associated with rotation and shape are expected to be small considering the 2° threshold we used to trigger robotic couch correction. Using a spherical model of a large prostate (2.5 cm radius, 65 mL volume), a 2° rotation around its center results in only sub‐millimeter deviations of points along the surface. Deviations of this magnitude are captured within the margins we have determined by our translational van Herk analysis and are consistent with a previous investigation examining the impact of prostate rotations during electromagnetically guided prostate radiotherapy.^24^ Additional errors associated with the treatment planning process related to MRI and CT co‐registration may be as high as 2 mm,^25^ and the potential for fiducial migration must also be considered. We attempted to minimize these errors by using stranded gold fiducials separated by a rigid titanium wire. This reduces the likelihood of fiducial migration and assists with rotational and translational alignment of the planning MRI to the planning CT by improving fiducial visualization between the datasets. Additionally, all prostate volumes were delineated using the planning MRI and verified on the CT to minimize any contouring errors.

Another limitation of our study results from the lack of continuous prostate tracking. Stereoscopic fiducial x‐ray verifications were performed every 90° of arc therapy, which corresponded to approximately every 20 s during treatment. It is possible that large prostate excursions occurred between x‐ray verifications and were unaccounted for in our dataset. However, these would be limited to transient excursions, as persistent sources of motion would have been captured during subsequent fiducial verifications. Furthermore, continuous optical and thermal surface tracking was used throughout treatment to account for patient motion exceeding normal cyclic respiratory patterns, which were empirically limited to 5 mm.

## 5 CONCLUSIONS

Spatial and temporal localization of the prostate plays a critical role in the treatment of prostate cancer using radiation therapy. The ability to accurately determine the position of the prostate and account for intrafraction motion impacts the magnitude of required treatment margins around the prostate and has the potential to mitigate treatment‐associated toxicity. Using a combined intrafraction stereoscopic x‐ray fiducial monitoring system together with continuous surface monitoring and a 6DoF robotic couch, we found that prostate CTV to PTV margins necessary to account for intrafraction motion during prostate SBRT may be as little as 1.23–1.73 mm. Importantly, this does not account for other sources of treatment error, which must be considered when determining final CTV to PTV treatment margins.

## AUTHOR CONTRIBUTIONS

Matthew Worth, Julie Lo, Daniel C. Brancato, and Thomas P. Kole contributed to the composition of the manuscript. Matthew Worth and Thomas P. Kole conducted the data collection and analysis.

## CONFLICT OF INTEREST STATEMENT

Daniel C. Brancato, Julie Lo, and Thomas P. Kole receive speaker honoraria from Brainlab.

## Data Availability

The data that support the findings of this study are available on request from the corresponding author. The data are not publicly available due to privacy or ethical restrictions.
